# Sex differences in volume overload in skinned fibers

**DOI:** 10.1186/s12872-016-0370-8

**Published:** 2016-10-14

**Authors:** C. Bening, K. Hamouda, R. Leyh

**Affiliations:** 1 Department of Thoracic, Cardiac and Thoracic Vascular Surgery, Medical Centre of the University Hospital Würzburg, Oberdürrbacherstrasse 6, 97080 Würzburg, Germany; 2 Department of Cardiothoracic and Vascular Surgery, University Hospital Mainz, Mainz, Germany

**Keywords:** Calcium sensitivity, pCa, Force relationship, Skinned fibers

## Abstract

**Background:**

The impact of sex on cardiac morphology and function in chronic volume overload has been described in detail. However, the relation between sex and contractile properties at the actin-myosin level has not been well defined. Therefore, we evaluated the influence of sex on the contractile capacities of patients with chronic volume overload.

**Methods:**

In 36 patients (18 males, 65 ± 9 years; 18 females, 65 ± 13 years) scheduled for elective mitral valve surgery due to severe mitral regurgitation (MR) with preserved left ventricular function, right auricle samples were obtained prior to extracorporal circulation. The fibers were prepared and skinned and exposed to a gradual increase in the calcium concentration (from pCa of 6.5–4.0) for calcium-induced force-developing measurements. Calcium sensitivity was also measured and recorded.

**Results:**

The pCa-force relationship of the fibers obtained from males and females was significantly different, with the force values of the female fibers greater than those of male fibers at maximum calcium concentrations (pCa of 4.0: 3.6 ± 0.3 mN versus 3.2 ± 0.4 mN, *p* 0.02) and pCa of 4.5 2.6 ± 0.6 versus 2.0 ± 0.5, *p* 0.002). In contrast, the force values of female fibers were lower at mean calcium concentrations compared to those of male fibers (at 5.5 and pCa of 6.0: 1.0 ± 0.3 mN versus 1.2 ± 0.5 mN, *p* 0.04; 0.61 ± 0.05 versus 0.88 ± 0.09, *p* 0.04).

Calcium sensitivity was observed at pCa of 5.0 in females and pCa of 4.5 in males.

**Conclusion:**

This study demonstrated that female fibers from patients exposed to chronic volume overload developed higher force values at a given calcium concentration compared to fibers from male patients. We assume that female patients might tap the full force potential, which is required when exposed to the highest calcium concentrations in our experimental cycle. The calcium sensitivity among genders was significantly different, with the results suggesting that males have higher calcium sensitivity and might compensate for lower force values at maximal calcium concentrations by a higher affinity for calcium. Hence, female patients with MR seem to work more “energy efficient”.

## Background

Chronic volume overload leads to various adaption mechanisms, such as increased ventricular mass and end-diastolic volume [1]. Several studies of rodents have shown that chronic volume overload led to extensive hypertrophy and marked dilatation in male rats but not in female rats [2]. Furthermore, female rats exposed to chronic volume overload did not exhibit adverse ventricular remodeling and showed reduced mortality compared to male rats [3]. Thus, classical signs of heart failure are missing in female rats with induced volume overload. In humans, chronic mitral regurgitation (MR) caused left ventricular dilation, a study showed that the end-diastolic dimensions increased, whereas chronic MR led to eccentric hypertrophy [4].

Calcium plays a major role in the cardiac contraction cycle. Evidence has long existed for sex differences in intrinsic cardiac contractile properties [5], positive inotropic responses to Ca2+ [6], Ca2+ channels in the heart [7], and hormone responsiveness [5, 8]. In accordance with these results, recent studies suggested that sex hormones may attenuate remodeling processes in chronic volume overload and that sex may influence the development of cardiac pathologies in chronic volume overload [2, 3, 9–12]. It is not known whether this pathology affects contractile properties at the level of the contractile apparatus or whether the influence of sex hormones differs between males and female patients. However, some recent animal studies reported that sex influenced ventricular remodeling induced by chronic volume overload [5, 6]. For example, Gardner and coworkers concluded that male rats with induced volume overload developed significant dilatation of left ventricles after 8 weeks, whereas female rats did not [2, 3]. Thus, classical signs of heart failure are missing in female rats with induced volume overload. These data were supported by Dent and coworkers, who showed that volume overload in mice led to a greater increase in cardiac muscle mass and wall thickness than in female mice, without any changes in the ventricular diameter, whereas the ventricular dimensions increased and left ventricular end-diastolic pressure in male mice [11, 12]. This demonstrates that sex modifies ventricular remodeling in rodents exposed to volume overload.

There are limited data in the literature on sex differences in the effect of chronic volume overload on contractility in humans with severe MR. Therefore, the aim of the present study was to evaluate the impact of chronic volume overload on the contractility of right auricle tissue of female and male patients scheduled for elective mitral valve surgery.

## Methods

The ethics committee of the Medical Association of Rheinhessen approved this study. The experiments were carried out in the experimental laboratory of the Department of Cardiothoracic and Vascular Surgery of the University Hospital of Mainz.

All the patients were informed and gave written consent for the use of intraoperative resected tissue in further research. All the experiments were carried out according to the Declaration of Helsinki.

Eighteen female and 18 male patients with pure severe mitral valve insufficiency who were scheduled for elective mitral valve surgery were included in the study. The exclusion criteria were aged <18 years, emergency surgery, atrial fibrillation, primary pulmonary hypertension, other valve lesions, coronary artery disease, an Ejection Fraction of <50 %, and endocarditis.

Echocardiographic data were obtained preoperatively from echocardiographic examinations performed in our hospital. We defined left atrial dilatation as >38 mm ap (in females) and >40 mm ap in males and left ventricular dilatation (m-mode measurements) as >53 mm (in females) and >59 mm (in males). Dilatation of the mitral annulus was defined for males/females in end-systolic apical four-chamber view more than 3.8 cm/3.3 cm (end-diastolic apical four-chamber view more than 4.1 cm/3.6 cm) [12].

### Skinned fiber preparation

Right auricle tissue was taken from the 36 patients prior to implementation of extracorporal circulation and prepared for skinned fiber experiments. The process of skinned fiber preparation has been described in detail elsewhere [8–10]. Briefly, the fibers were collected in the operating theater after resecting the right auricle. Using the no-touch-technique, the auricular tissue was transferred to an ice-cooled vial (4 °C) filled with an oxygenated cardioplegic solution (1000 ml of Krebs-Henseleit solution in mmol/L of 118.07 NaCl, 11.1 C_6_H_12_O_6_ + H_2_O, 4.7 KCK, 25 NaHCO_3_, 1.2 KH_2_PO_4_, 1.2 MgSO_4_ + 7 H_2_O, and 1.8 CaCl_2_ + 2H_2_O). From this solution, 100 ml were removed and 30 mmol/L of an ATP-sensitive potassium canal inhibitor, C_4_H_2_NO_2_ (2,3-butanedione monoxime), were added. The auricular was then placed in a dish filled with 20 ml of a preparation solution (4 °C) (amounts in mM: C_3_H_4_N_2_ 68.08, NaN_3_ 65.01, C_14_H_24_N_2_O_10_ 380.4, C_4_H_10_O_2_S_2_.154.3, MgCl_2_ × 6 H_2_O 203.3, C_10_H_14_N_5_O_13_ P_3_ Na_2_ 605.2). Subsequently, the muscle bundles were resected from the auricle and placed in a test tube containing 9900 μl of the preparation solution, including 1 % Triton-X-100 (100 μl), for 24 h at 4 °C on a shaking device. The fibers were skinned after being rolled for 24 h on a special rotation device to remove membrane-dependent properties [9]. Following this procedure. the fiber bundles were prepared for the experimental setup.

The experimental setup consisted of a specially designed “gradient measurement device,” in which the concentration inside the perfusion chamber was automatically increased stepwise during the experiment (Scientific Instruments, Heidelberg, Germany). The device consists of two pumps, which transports and withdraws a specific amount of the solution in and out of a perfusion chamber. In this perfusion chamber, the skinned fiber was fixed between two forceps and immersed in the solution. As the calcium-containing solution was transported to the perfusion chamber, the concentration was increased in a stepwise manner. When the skinned fiber started to contract, the resulting force-development curve was recorded by the attached computer system. Of course, to achieve a special calcium concentration, which is calculated by the attached computer system, several parameters (the concentration of EGTA, free calcium concentration, tubing volume, volume of the perfusion chamber etc.) have to be entered in the computer system before starting the experiments. Thus, the concentration of calcium can be calculated precisely (Gradient Program, Scientific Instruments, Heidelberg).

For the experimental set-up, the fibers were cut in strips of 2–2.5 mm × 0.3 mm and fixed between two forceps in a cuvette, which served as a perfusion chamber. To achieve steady state conditions, the cuvette was perfused with a relaxation solution (contents in mM: C_3_H_4_N_2_ 68.08, C_4_H_8_N_3_O_5_PNa_2_ + 4 H_2_O 327.2, NaN_3_ 65.01, C_14_H_24_N_2_O_10_ 380.4, MgCl_2_ 203.3, C_4_H_10_O_2_S_2_.154.2, and C_10_H_14_N_5_O_13_ P_3_ Na_2_ 605.2) and 400 U/ml of creatine kinase (Boehringer, Mannheim, FRG). To induce contraction, solution calcium and CaCl_2_ (147.02 mM) were added. The free calcium concentration was obtained by mixing the relaxation and contraction solution in appropriate proportions. The desired calcium concentrations were calculated by a computer program (Scientific Instruments, Heidelberg, Germany), following the equation of Fabiato and Fabiato and given as pCa (−log of free [Ca]_+_) until steady state conditions were observed. Afterwards, the fibers were carefully prestretched to 20 mg to achieve optimal overlapping of contractile elements (according to a cardiomyocyte sarcomere length of 2 μm). After achieving a steady state, the fibers were exposed to the contraction solution. A computer program calculated the desired calcium concentrations (i.e., pCa 6.5 until 4.0) (Scientific Instruments Heidelberg, Germany).

The fibers were exposed to a continuous increase in the calcium concentration, starting at pCa 6.5 (lowest calcium concentration) and ending at pCa 4.0 (highest calcium concentration). The rising calcium concentration and responding force development were simultaneously recorded and sampled on the attached computer. Three fibers from each patient were exposed to cycles of increasing calcium concentrations. Thus, the final analysis consisted of 54 data sets for female fibers and 54 data sets for male fibers.

### Statistical analysis

The statistical analysis was performed using SPSS software 13.0 for PC (Analytical Software, Chicago, IL, USA). Values are expressed as the mean ± S.D, and numbers of patients are expressed as percentages. The Shapiro–Wilk test was used to verify normal statistical distributions. The Welch test was used to evaluate statistically significant differences between males and females in RAP, wedge, LVESV, height, weight, and BMI. The Wilcoxon rank-sum test was used to verify statistically significant differences in the force values of pCa 6.5–4.0, TAPSE, age, and EF. A Chi square test was used to evaluate differences in the prevalence of the dilatation of the mitral valve ring, LA and LV dilatation, and grade of TR and MR. Statistical significance was determined using an alpha level of < 0.05.

## Results

**Fig. 1:**
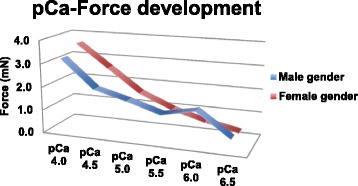
Comparison of female and male pCa force development in patients with volume overload

The force values of skinned fibers from females were higher than those of male fibers at the highest calcium concentration (pCa of 4.0: 3.6 ± 0.3 mN versus 3.2 ± 0.4 mN, *p* 0.02; pCa of 4.5 2.6 ± 0.6 versus 2.0 ± 0.5, *p* 0.002) and lower than those of male fibers at mean calcium concentrations (pCa of 5.5 and pCa of 6.0: 1.0 ± 0.3 mN versus 1.2 ± 0.5 mN, *p* 0.04; 0.61 ± 0.05 versus 0.88 ± 0.09, *p* 0.04, see Fig. 1). At mean steps of calcium concentrations (pCa 5.0), the differences subsided (females 1.6 ± 0.5 mN versus 1.63 ± 0.9, *p* 0.1).

Calcium sensitivity, given as the calcium concentration at which the half maximal concentration was achieved, occurred at pCa 5.0 in males and 4.5 in females. Hence, the males were more sensitive to calcium than females (*p* 0.03, Tables 1 and 2).

**Table 1:** Summary of clinical data of the patients

Gender	Female	Male
Number	18	18
Age (years)	65 ± 9	65 ± 13
Height (cm)	164 ± 8	174 ± 9
Weight (kg)	69 ± 12	76 ± 13
BMI	25 ± 5	25 ± 3
Left Atrium Dilatation (number of patients)	7 (35 %)	9 (45 %)
Left Ventricle Dilatation (number of patients)	4 (20 %)	4 (20 %)
Mitral Valve Annulus Dilatation (number of patients)	3 (15 %)	11 (55 %)
Prolapse of Mitral Valve	10 (50 %)	9 (45 %)
Ejection Fraction (%)	46 ± 11	54 ± 11
Mean grade of MR	3.4 ± 1.1	4 ± 0.3
Enddiastolic Septum thickness (mm)	15 ± 5 mm	11 ± 4.1 mm
Mean PCWP (mmHg)	25.5 ± 4	19 ± 7
Mean mPAP (mmHg)	45 ± 20	36 ± 9
Mean RAP (mmHg)	6.8 ± 2.2	9.5 ± 2.7
TAPSE	21.3 ± 3.7	19.3 ± 4.2
LVESV (ml)	43.9 ± 11.5	51.3 ± 11.8
Grade of TR	1 ± 0.2	1 ± 0.1

**Table 2:** Summary of pCa-force values

	Male patients	Female patients	*p*
pCa 4.0	3.2 ± 0.4 mN	3.6 ± 0.3 mN	0.02
pCa 4.5	2.0 ± 0.5 mN	2.6 ± 0.6 mN	0.002
pCa 5.0	1.63 ± 0.9 mN	1.6 ± 0.5 mN	0.1
pCa 5.5	1.2 ± 0.5 mN	1.0 ± 0.3 mN	0.04
pCa 6.0	0.88 ± 0.09 mN	0.61 ± 0.05 mN	0.04
pCa 6.5	0.51 ± 0.1 mN	0.32 ± 0.3 mN	0.1
pCa^++^_50_	5.0	4.5	0.03

Comparing the clinical data, we observed that dilatation of the mitral valve annulus occurred significantly more often in the female group (55 versus 15 %, *p* 0.01). There was no significant sex difference in the prevalence of left atrial dilation and left ventricular dilatation. Female gender differed significantly in the end-diastolic septum thickness (11 mm versus 15 mm, *p* 0.04), which was smaller in female gender. Additionally, the females were significantly shorter than the males (164 ± 8 versus 174 ± 9, *p* 0.003). However, the BMI of the two groups was similar. The RAP (6.8 ± 2.2 versus 9.5 ± 2.7, *p* 0.003) was significantly lower in females than males (25.5 ± 4 versus 19 ± 7, *p* 0.001, Table 3). The wedge pressure (25.5 ± 4 mmHg versus 19 ± 7 mmHg, *p* 0.001) and mean pulmonary artery pressure (PAP) (45 ± 20 mmHg versus 36 ± 9 mmHg, n.s.) were higher in females than in males. In addition, the TAPSE (21.3 ± 3.7 versus 19.3 ± 4.2, n.s.) was elevated, and LVEF (43.9 ± 11.5 versus 51.3 ± 11.8, *p* 0.04) was slightly decreased.

**Table 3:** Summary of the results

Gender	Female	Male	*p*
Number	18	18	
Age (years)	65 ± 9	65 ± 13	n.s
Height (cm)	164 ± 8	174 ± 9	0.003
Weight (kg)	69 ± 12	76 ± 13	n.s
BMI	25 ± 5	25 ± 3	n.s
Left Atrium Dilatation (number of patients)	9 (50 %)	7 (38 %)	n.s
Left Ventricle Dilatation (number of patients)	4 (22 %)	8 (44 %)	n.s
Mitral Valve Annulus Dilatation (number of patients)	3 (16 %)	11 (61 %)	0.01
Prolapse of Mitral Valve	10 (55 %)	9 (50 %)	n.s
Ejection Fraction (%)	46 ± 11	54 ± 11	n.s
Mean grade of MR	3.4 ± 1.1	4 ± 0.3	n.s
Enddiastolic Septum thickness (mm)	15 ± 5 mm	11 ± 4.1 mm	0.04
Mean PCWP (mmHg)	25.5 ± 4	19 ± 7	0.001
Mean mPAP (mmHg)	45 ± 20	36 ± 9	n.s
Mean RAP (mmHg)	6.8 ± 2.2	9.5 ± 2.7	0.003
TAPSE	21.3 ± 3.7	19.3 ± 4.2	n.s.
LVESV (ml)	43.9 ± 11.5	51.3 ± 11.8	0.04
Grade of TR	1 ± 0.2	1 ± 0.1	0.5

## Discussion

In this study, we observed sex differences in the contractile performance of patients with severe MR. Skinned fibers from females achieved more force at higher supra-normal calcium concentrations (pCa of 4.0 and pCa of 4.5). In contrast, male fibers developed higher forces at lower calcium concentrations (pCa of 5.0–6.5), which included the “physiological concentration” at pCa of 5.0–6.0. The calcium sensitivity of the male and female fibers was similar. We propose that females save force capacities, which can be mobilized at higher force values, whereas males tap the full force capacity at physiological concentrations of pCa of 5.0 and 6.0. However, it is unclear how female fibers could develop increased contractibility at higher calcium concentrations. Thus, it is uncertain whether force fatigue. On the other hand, clinical signs, such as wedge pressure, mean PAP, TAPSE, and LVEF might give some evidence for a reduced biventricular function, which was observed in females, compared to males. Although there is controversy in the literature on the force values of healthy male and female skinned fibers (i.e., whether they are higher in females or males), all the previous studies were performed in rats or other animal models [1–3, 5–7, 9, 10].

In common with the findings of the present study, Schwertz observed increased forces in female skinned human rat atria at high extracellular calcium in the presence of tetanic contraction and greater force and a higher maximal rate of contraction in females compared to males [8]. Another study also reported that the calcium sensitivity of female rats seemed to be higher than that of male rats [6].

In the presence of volume overload, Dent found sex-specific differences: Females developed greater increase in cardiac mass, but the rates of pressure development (+dP/dt) and decay (−dP/dt) were depressed and left ventricular end-diastolic pressure increased in male rats at 16 weeks post-AV shunt. Decreased LV internal diameters, as well as depressed fractional shortening, occurred in males, whereas increases in posterior wall thickness were seen in female rats at 16 weeks post-AV shunt [11, 12]. Therefore, sex-specific remodeling processes seem to occur, at least in rodents. In the present study, the pressure values (wedge and PAP) of females increased more often than those of males, but they had decreased RAP values. Furthermore, the LVEF was slightly reduced in females compared to males. Thus, the observations are somewhat similar to those reported by Dent. Calcium sensitivity was different among genders, males needed less calcium to achieve half maximal activation. This means, they are more sensitive to calcium compared to females. Wattanapermpool and coworkers presented similar results, with a loss of estrogen by ovariectomy increasing the sensitivity of rats to calcium [13]. Their results are in accordance with the findings in the present study of reduced calcium sensitivity in fibers obtained from female patients.

According to the difference in cardiac properties, we observed gender-related differences in the clinical picture of mitral valve regurgitation scheduled for elective mitral valve surgery due to MR. We observed more mitral annulus dilatation in females but no significantly higher incidence of prolapse, flail leaflet, or chorda rupture in one of the groups. However, prolapse and LA dilatation were more common in the female group. These findings are accordance with those in the literature [14].

In the present study, although there were no differences in the preoperative clinical presentation in terms of pathological findings in the mitral valve, sex-specific cardiac remodeling can be assumed, as female patients present more frequently with left atrial dilatation, and male patients present more often with left ventricular dilatation. However, this was not significant, but might be an explanation for reduced force capacity at the level of the atria because right heart function parameters, such as TAPSE, were also slightly increased in females. In the literature, there are only a few studies on the sex-specific clinical presentation in humans with severe MR.

Several animal studies have provided evidence for differences in the remodeling process. Janicki et al. described a sex-specific remodeling process in a rodent model of induced volume overload [15]. In their study, female rodent hearts showed less chamber dilatation but more ventricular hypertrophy than male hearts, which showed significant dilatation and wall thinning [15]. Another rodent study demonstrated that the female heart was cardioprotected, developed concentric hypertrophy, and remained compensated [16]. The idea of sex-specific remodeling processes extends to the cellular level. Studies reported an increase in the cardiomyocyte cross-sectional area (parallel sarcomeres) and length (in-series sarcomeres) after volume overload in female rats but no such increases in male rats [17, 18]. This might explain the possibility of females to recruit further force when exposed to even higher calcium concentrations. Further studies should be conducted to determine whether such a compensatory mechanism exists and its clinical relevance.

In our opinion, these findings could have clinical relevance for on the timing of interventions in patients presenting with severe mitral valve regurgitation. To date, the guidelines on surgical treatment for asymptomatic severe MI have relied solely on the level of LV dilatation, end-systolic diameter, EF, and presence of atrial fibrillation and/or pulmonary hypertension [19]. If we assume the existence of a sex-specific remodeling process, it is possible that female patients are referred too late for mitral valve surgery because the first response to overload in females is ventricular hypertrophy and an increase in LV mass rather than ventricular dilatation. The aforementioned hypothesis is supported by data from the literature showing that the short- and long-term survival of females who undergo surgery for MR is worse than that of male patients [20].

### Limitations of the study

The advantage of the preparation used in the present study is the independency of membrane-dependent processes, as pharmacological influences (pre- and perioperative medications) can be excluded due to the wash-out process [9]. Nevertheless, this study has some limitations. First, the duration of asymptomatic MR may have influenced the results, as we do had no data on the duration of presurgical treatment for MR. Although the potential effect of pharmacological treatment can be likely excluded by the “skinning fiber model”, Wankerl showed that an effect of various kinds of cardiac disease (independent of the disease duration) could solely be shown in human atrial tissue, since in ventricular tissue differences in cardiac sensitivity are no more existent [6]. Second, the mechanism of skinning, as well as demembranizing the fibers, could have influenced the contractile ability of the fibers and influenced the results. Third, a selection bias could have influenced the data due to the small sample size.

## Conclusions

These preliminary data suggest that female patients are better able than males to tolerate volume overload, as higher force values were recorded. The force capacity of male fibers significantly reduced. The reduction in the force capacity could be due to various factors, such as a sex-specific response to volume overload, global cardiomyocyte remodeling due to volume overload, or changes in calcium handling. The apparent ability of females to tolerate volume overload are in accordance with the clinical picture of late diagnosis and treatment of female patients presenting with severe MR.

The existing sex-specific differences in postoperative outcomes, in addition to our experimental findings, seem to justify a re-evaluation of the management of mitral valve disease in female patients. Further studies with a larger number of patients are needed to support these observations and to elucidate the clinical relevance of these results.

## Abbreviations

ATP: Adenosintriphosphat
BMI: Body mass index
CAD: Coronary artery disease
EF: Ejection fraction
LA: Left atrium
LV: Left ventricle
LVEDD: Left ventricular enddiastolic diameter
MR: Mitral regurgitation
NYHA: New York Heart Association
PAP: Pulmonary artery pressure
pCa: Calcium concentration
pCa50**: Calcium sensitivity
RA: Right atrium
RAP: Right atrial pressure
TAPSE: Tricuspid annular plane excursion
TR: Tricuspid regurgitation

## References

[1] Brower GL, Henegar JR, Janicki JS (1996). Temporal evaluation of left ventricular remodeling and function in rats with chronic volume overload. Am J Physiol.

[2] Gardner JD, Brower GL, Janicki JS (2002). Gender differences in cardiac remodeling secondary to chronic volume overload. J Card Fail.

[3] Gardner JD, Murray DB, Voloshenyuk TG, Brower GL, Bradley JM, Janicki JS (2010). Estrogen attenuates chronic volume overload induced structural and functional remodeling in male rat hearts. Am J Ohysiol Heart Circ Ohysiol.

[4] Zile MR, Tomita M, Nakano K, Mirsky I, Usher B, Lindroth J, Carabello BA (1991). Effects of left ventricular volume overload produced by mitral regurgitation on diastolic function. Am J Physiol.

[5] Capasso JM, Remily RM, Smith RH, Sonnenblick EH (1983). Sex differences in myocardial contractility in the rat. Basic Res Cardiol.

[6] Wang SN, Wyeth RP, Kennedy RH (1998). Effects of gender on the sensitivity of rat cardiac muscle to extracellular Ca2+. Eur J Pharmacol.

[7] Ishii K, Kano T, Ando J (1988). Sex differences in [3H]nitrendipine binding and effects of sex steroid hormones in rat cardiac and cerebral membranes. Jpn J Pharmacol.

[8] Schwertz DW, Vizgirda V, Solaro RJ, Piano MR, Ryjewski C (1999). Sexual dimorphism in rat left atrial function and response to adrenergic stimulation.

[9] Wankerl M, Böhm M, Morani I, Ruegg JC, Eichhorn M, Erdmann E (1990). Calcium sensitivity and myosin light chain pattern of atrial and ventricular skinned cardiac fibers from patients with various kinds of cardiac disease. J Moll Cell Cardiol.

[10] Ayaz O, Howlett SE (2015). Testosterone modulates cardiac contraction and calcium homeostasis: cellular and molecular mechanisms. Biol Sex Differ.

[11] Dent MR, Tappia PS, Dhalla NS (2010). Gender Differences in cardiac dysfunction and remodeling due to volume overload. J Card Fail.

[12] Dent MR, Tappia PS, Dhalla NS (2010). Gender differences in apoptotic signaling in heart failure due to volume overload. Apoptose.

[13] Wattanapermpool J (1998). Increase in calcium responsiveness of cardiac myofilament activation in ovariectomized rats. Life Sci.

[14] Avierinos JF, Inamo J, Grigioni F, Gersh B, Shub C, Enriquez-Sarano M (2008). Sex differences in the morphology and outcomes of mitral valve prolapse: a cohort study. Ann Intern Med.

[15] Janicki JS, Spinale FG, Levick SP (2013). Gender differences in Non-ischemic myocardial remodeling: are they Due to estrogen modulation of cardiac mast cells and/or membrane Typ I matrix metalloproteinase. Pflugers Arch.

[16] Du Y, Plante E, Janicki JS, Brower GL (2010). Temporal evaluation of cardiac myocyte hypertrophy and hyperplasia in male rats secondary to chronic volume overload. Am J Pathol.

[17] Sofia RR, Serra AJ, Silva JA, Antonio EL, Manchini MT, Oliveira FA, Teixeira VP, Tucci PJ (2014). Gender-based differences in cardiac remodeling and ILK expression after myocardial infarction. Arq Bras Cardiol.

[18] Liu Z, Hilbelink DR, Crockett WB, Gerdes AM (1991). Regional changes in hemodynamics and cardiac myocyte size in rats with aortocaval fistulas. 1. Developing and established hypertrophy. Circ Res.

[19] Vahanian A (2012). Guidelines on the management of valvular heart disease (version 2012). Joint Task Force on the Management of Valvular Heart Disease of the European Society of Cardiology (ESC); European Association for Cardio-Thoracic Surgery (EACTS). Eur Heart J.

[20] Seeburger J, Eifert S, Pfannmüller B, Garbade J, Vollroth M, Misfeld M, Borger M, Mohr FW (2013). Gender differences in mitral valve surgery. Thorac Cardiovasc Surg.

